# Correction: Context-dependent deposition and regulation of mRNAs in P-bodies

**DOI:** 10.7554/eLife.41300

**Published:** 2018-08-23

**Authors:** Congwei Wang, Fabian Schmich, Sumana Srivatsa, Julie Weidner, Niko Beerenwinkel, Anne Spang

Wang C, Schmich F, Srivatsa S, Weidner J, Beerenwinkel N, Spang A. 2018. Context-dependent deposition and regulation of mRNAs in P-bodies. *eLife* **7**:e29815. doi: 10.7554/eLife.e29815.

Published 3, January 2018

Recently, we identified a mistake in the read count file for the total RNA seq data. The raw sequencing data had been inadvertently mapped onto the wrong strand. We corrected the mapping and deposited the corrected file on GEO (GSE76444_TotalmRNA_counts_raw_txt.gz). The numbers presented in the Venn diagrams in Figure 1D and the total RNAseq hits list in Supplementary File 3 were corrected. The analyses of the total RNAseq data in Figure 1—figure supplement 1D-1G were performed with the new hit list and amended. We have updated the text relating to Figure 1D to”mRNAs that were upregulated upon glucose starvation, as determined by total RNA-Seq, were by and large not enriched in the corresponding fraction of the P-body components. Less than 16% overlap was observed between mRNAs generally upregulated in stress response and those pulled down by P-body components (Figure 1D). This exclusive enrichment was less pronounced for the hyperosmotic stresses. The new Figure 1, Figure 1—figure Supplement 1 and Supplementary file 3 are provided with this correction. The corrections do not change any of the conclusions of the manuscript. We apologize for the mistake.

Supplementary file 3.

The corrected Figure 1 is shown here:

**Figure 1:**
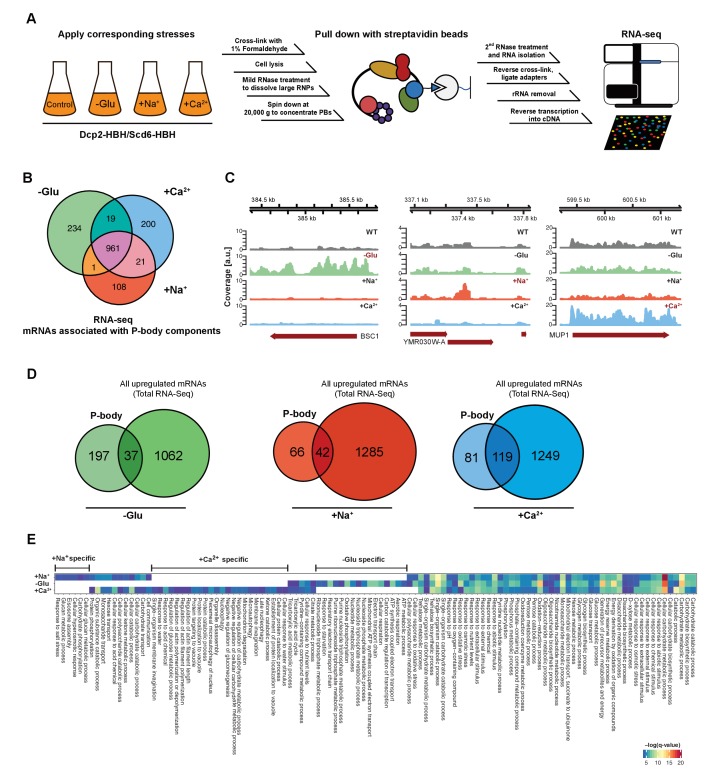


The originally published Figure 1 is also shown for reference:

**Figure 2:**
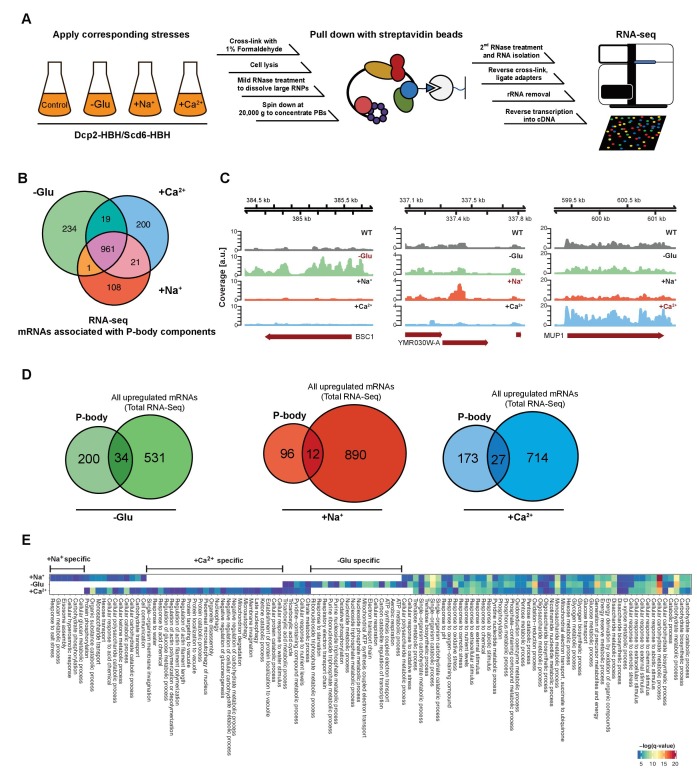


The corrected Figure 1—figure supplement 1 is shown here:

**Figure 3:**
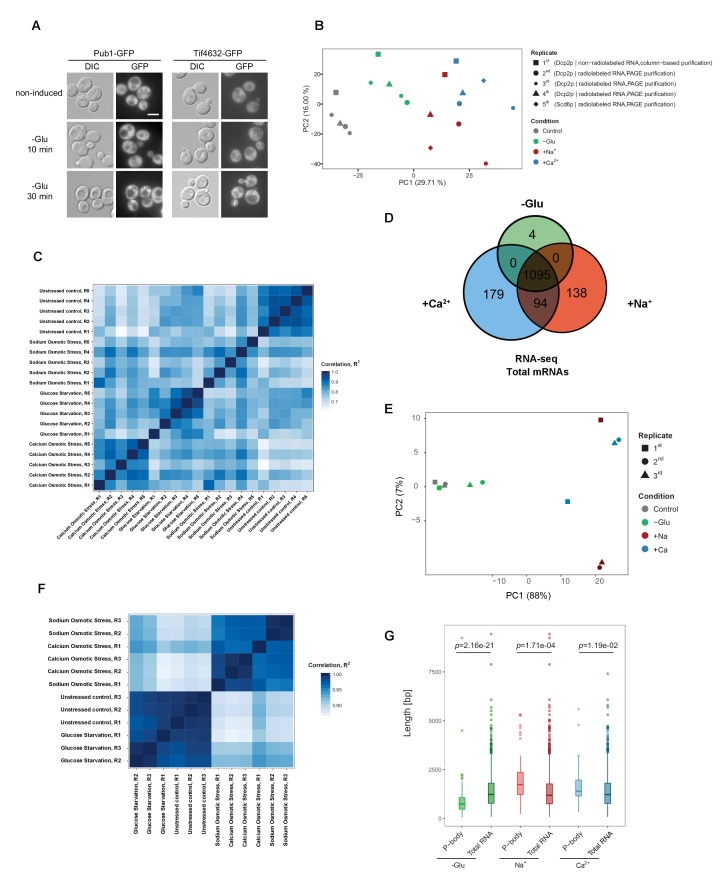


The originally published Figure 1—figure supplement 1 is also shown for reference:

**Figure 4:**
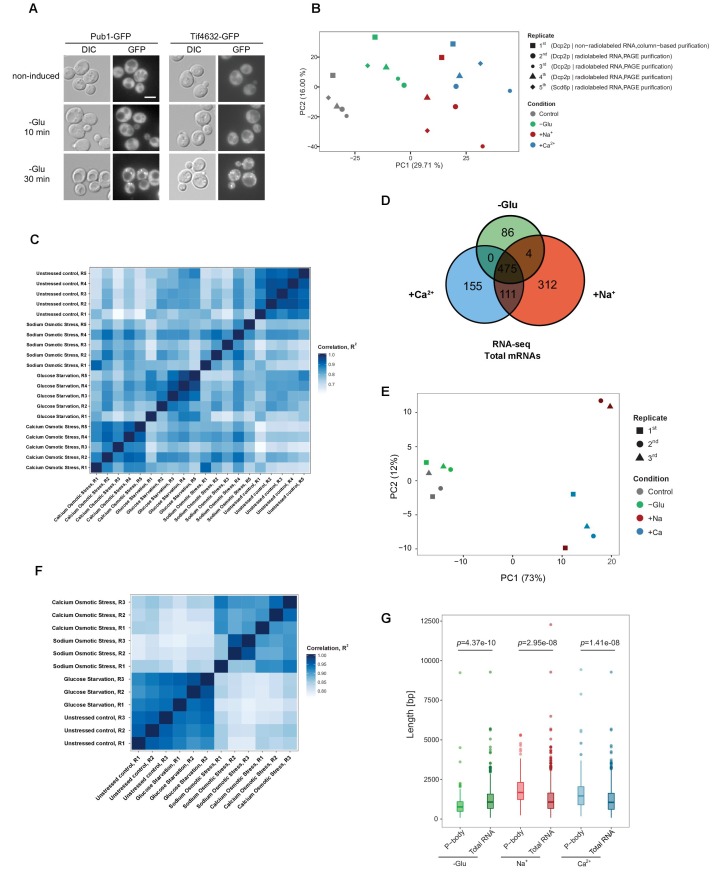


The article has been corrected accordingly.

