# Supplementary material for: Context-dependent deposition and regulation of mRNAs in P-bodies
Source: eLife. 2018 Jan 3;7:e29815. doi: 10.7554/eLife.29815 (PMC5752201; doi:10.7554/eLife.29815)
Supplement: Supplementary file 1. [file elife-29815-supp1.docx]

**Supplementary File 1. Experimental Design.**

| Condition | Gel Label | Batch | Replicate |
| --- | --- | --- | --- |
| Unstressed control | non-radiolabeling | March | R1 |
| Glucose Depletion | non-radiolabeling | March | R1 |
| Sodium Osmotic Stress | non-radiolabeling | March | R1 |
| Calcium Osmotic Stress | non-radiolabeling | March | R1 |
| Unstressed control | radiolabeling | March | R2 |
| Glucose Depletion | radiolabeling | March | R2 |
| Sodium Osmotic Stress | radiolabeling | March | R2 |
| Calcium Osmotic Stress | radiolabeling | March | R2 |
| Unstressed control | radiolabeling | May | R3 |
| Glucose Depletion | radiolabeling | May | R3 |
| Sodium Osmotic Stress | radiolabeling | May | R3 |
| Calcium Osmotic Stress | radiolabeling | May | R3 |
| Unstressed control | radiolabeling | August | R4 |
| Glucose Depletion | radiolabeling | August | R4 |
| Sodium Osmotic Stress | radiolabeling | August | R4 |
| Calcium Osmotic Stress | radiolabeling | August | R4 |
| Unstressed control | radiolabeling | October | R5 |
| Glucose Depletion | radiolabeling | October | R5 |
| Sodium Osmotic Stress | radiolabeling | October | R5 |
| Calcium Osmotic Stress | radiolabeling | October | R5 |
