# Supplementary material for: Context-dependent deposition and regulation of mRNAs in P-bodies
Source: eLife. 2018 Jan 3;7:e29815. doi: 10.7554/eLife.29815 (PMC5752201; doi:10.7554/eLife.29815)
Supplement: Supplementary file 4. [file elife-29815-supp4.docx]

**Supplementary File 4. List of primers used in this study.**

| Primer ID | Designation | Sequence |
| --- | --- | --- |
| CK059 | Dcp2 S2 C-tagging-forward | TTCATTTACAGTGTGTCTATAAAACGTATAACACTTATTCTTTCAATCGATGAATTCGAGCTCG |
| CK060 | Dcp2 S3 C-tagging-reverse | GGAACTTCAGGGTCTAATGAATTATTAAGCATTTTGCATAGGAAGCGTACGCTGCAGGTCGAC |
| CW156 | 3' adapter (RA3) | TGGAATTCTCGGGTGCCAAGG |
| SH138 | 5' adapter (RA5) | GUUCAGAGUUCUACAGUCCGACGAUC |
| CW157 | Illumina Indexing Primer (RPI1) | CAAGCAGAAGACGGCATACGAGATCGTGATGTGACTGGAGTTCCTTGGCACCCGAGAATTCCA |
| CW158 | Illumina Indexing Primer (RPI2) | CAAGCAGAAGACGGCATACGAGATACATCGGTGACTGGAGTTCCTTGGCACCCGAGAATTCCA |
| CW159 | Illumina Indexing Primer (RPI3) | CAAGCAGAAGACGGCATACGAGATGCCTAAGTGACTGGAGTTCCTTGGCACCCGAGAATTCCA |
| CW160 | Illumina Indexing Primer (RPI4) | CAAGCAGAAGACGGCATACGAGATTGGTCAGTGACTGGAGTTCCTTGGCACCCGAGAATTCCA |
| CW244 | *PGK1*_1 FISH-forward | TTTGGAACACCACCCAAGAT |
| CW245 | *PGK1*_1 FISH-reverse | TAATACGACTCACTATAGGGAGCGTTTCTTTCACCGTTTGGT |
| CW246 | *PGK1*_2 FISH-forward | TGCGTTACCACATCGAAGAA |
| CW247 | *PGK1*_2 FISH-reverse | TAATACGACTCACTATAGGGAGCATCTTCCTTGGAAGCCTTG |
| CW248 | *PGK1*_3 FISH-forward | GGTGGTGGTATGGCTTTCAC |
| CW249 | *PGK1*_3 FISH-reverse | TAATACGACTCACTATAGGGAGCGAAGATGGAGTCACCGATT |
| CW250 | *PGK1*_4 FISH-forward | TTCTCTGCTGATGCCAACAC |
| CW251 | *PGK1*_4 FISH-reverse | TAATACGACTCACTATAGGGAGCAGCCAGCTGGAATACCTTC |
| CW252 | *PGK1*_5 FISH-forward | ACTGGTGGTGGTGCTTCTTT |
| CW253 | *PGK1*_5 FISH-reverse | TAATACGACTCACTATAGGGAGAAAGCAACACCTGGCAATTC |
| CW963 | *PGK1* qPCR-forward | TTGATTGACAACTTGTTGGA |
| CW964 | *PGK1* qPCR-reverse | CAGTGACAGTCTTGGTGTTG |
| CW278 | *ACT1*_1 FISH-forward | CTGAGGTTGCTGCTTTGGTT |
| CW279 | *ACT1*_1 FISH-reverse | TAATACGACTCACTATAGGGAG GCAAAACCGGCTTTACACAT |
| CW280 | *ACT1*_2 FISH-forward | TGTCACCAACTGGGACGATA |
| CW281 | *ACT1*_2 FISH-reverse | TAATACGACTCACTATAGGGAG TGTTCTTCTGGGGCAACTCT |
| CW282 | *ACT1*_3 FISH-forward | CGTTCCAATTTACGCTGGTT |
| CW283 | *ACT1*_3 FISH-reverse | TAATACGACTCACTATAGGGAG GGCCAAATCGATTCTCAAAA |
| CW284 | *ACT1*_4 FISH-forward | TCCACCACTGCTGAAAGAGA |
| CW285 | *ACT1*_4 FISH-reverse | TAATACGACTCACTATAGGGAG GAAGTCCAAGGCGACGTAAC |
| CW286 | *ACT1*_5 FISH-forward | ACATCGTTATGTCCGGTGGT |
| CW287 | *ACT1*_5 FISH-reverse | TAATACGACTCACTATAGGGAG CGGTGATTTCCTTTTGCATT |
| CW288 | *ACT1*_6 FISH-forward | TCCATCTTCCATGAAGGTCA |
| CW289 | *ACT1*_6 FISH-reverse | TAATACGACTCACTATAGGGAG CCACCAATCCAGACGGAGTA |
| CW961 | *ACT1* qPCR-forward | AGGTTGCTGCTTTGGTTATTG |
| CW962 | *ACT1* qPCR-reverse | CCGACGATAGATGGGAAGAC |
| CW560 | *BSC1*_1 FISH-forward | TCCGTATCATGGGTCAAGGA |
| CW561 | *BSC1*_1 FISH-reverse | TAATACGACTCACTATAGGGAGGAGGGACCGTTGACACCAA |
| CW562 | *BSC1*_2 FISH-forward & qPCR-forward | TCTGACGGTTGCACAGTTTG |
| CW563 | *BSC1*_2 FISH-reverse | TAATACGACTCACTATAGGGAGTGCCAAGTTTGCCAGTACTG |
| CW564 | *BSC1*_3 FISH-forward | CCTGGATTTTATTGGACCTACC |
| CW565 | *BSC1*_3 FISH-reverse | TAATACGACTCACTATAGGGAGGTAGTAATGGATGAAGACGAGGCTTT |
| CW566 | *BSC1*_4 FISH-forward | TTCATCAAGCGTAGCCGATA |
| CW567 | *BSC1*_4 FISH-reverse | TAATACGACTCACTATAGGGAGTGAAGTGAAACTGCTGAAAGGA |
| CW729 | *BSC1* qPCR-reverse | TGCCAAGTTTGCCAGTACTG |
| KT1009 | *BSC1* deletion forward | GTTGCTATCAATGGTTGTCCAAATTTGGATTTCAACTGGCACATGCAGCTGAAGCTTCGTACGC |
| KT1010 | *BSC1* deletion reverse | AGCGCTTGAACTAGTTGGGTCTTCTGAACTTGAACTTACTACTTAGCATAGGCCACTAGTGGATCTG |
| CW584 | *TPI1*_1 FISH-forward | AAACGGTTCCAAACAATCCA |
| CW585 | *TPI1*_1 FISH-reverse | TAATACGACTCACTATAGGGAGTGGAGGACAGATAACAACTTCG |
| CW586 | *TPI1*_2 FISH-forward | CGCCTACTTGAAGGCTTCTG |
| CW587 | *TPI1*_2 FISH-reverse | TAATACGACTCACTATAGGGAGCGGAGTGACCCAAAATAACC |
| CW588 | *TPI1*_3 FISH-forward & qPCR-forward | CAATTGAACGCTGTCTTGGA |
| CW589 | *TPI1*_3 FISH-reverse | TAATACGACTCACTATAGGGAGTCTGGAGTAGCAGCCAAACC |
| CW590 | *TPI1*_4 FISH-forward | GCCAGCGAATTGAGAATCTT |
| CW591 | *TPI1*_4 FISH-reverse | TAATACGACTCACTATAGGGAGATCGACATCAGCCTTGTCCT |
| CW734 | *TPI1* qPCR-reverse | TCTGGAGTAGCAGCCAAACC |
| CW766 | *RLM1*_1 FISH-forward | AAGAAGGCCCATGAACTATCC |
| CW767 | *RLM1*_1 FISH-reverse | TAATACGACTCACTATAGGGAGACGTGTTATTGGACCCCAGT |
| CW768 | *RLM1*_2 FISH-forward | CCGAGTGCACACATGAAGTT |
| CW769 | *RLM1*_2 FISH-reverse | TAATACGACTCACTATAGGGAGGCGTTCTCAGGATGACGTTT |
| CW770 | *RLM1*_3 FISH-forward | CCTTTAATGGTCGTCCTCCA |
| CW771 | *RLM1*_3 FISH-reverse | TAATACGACTCACTATAGGGAGGGTCCTTGAGGATGCATTTG |
| CW772 | *RLM1*_4 FISH-forward & qPCR-forward | AGGCCCCTTAACTCTCCAAA |
| CW773 | *RLM1*_4 FISH-reverse | TAATACGACTCACTATAGGGAGTAGAACCGTTAGGCGCATTT |
| CW774 | *RLM1*_5 FISH-forward | AATATTCCTGGCGGACCTTT |
| CW775 | *RLM1*_5 FISH-reverse | TAATACGACTCACTATAGGGAGTGTTGGTATTGCTGGGTTTG |
| CW776 | *RLM1*_6 FISH-forward | CGGGAAACACAAACAATCCT |
| CW777 | *RLM1*_6 FISH-reverse | TAATACGACTCACTATAGGGAGGTGCTGCTGATATTGCTGGA |
| CW1178 | *RLM1* qPCR-reverse | TAGAACCGTTAGGCGCATTT |
| CW1444 | *RLM1 pOMf* | TAAATTCTTTAATTTTT CGACTGATAT TTCAGGAGAA AAAAATTCAA GCAAAATATGCAGGTCGACAACCCTTAAT |
| CW1445 | *RLM1 pOMr* | AAAGAAGTTTCTTATGCTTGGAATATTCATACTGGTCAAATTTTTTGGTTTAGCGGCCGCATAGGCCACT |
| CW1027 | *ATP11*_1 FISH-forward | CTAATCAGCTGTCGCCCATT |
| CW1028 | *ATP11*_1 FISH-reverse | TAATACGACTCACTATAGGGAGGGGCTGGAAGAATAAAAACG |
| CW1029 | *ATP11*_2 FISH-forward | GGAAGAGGCTCAAAAACAAGG |
| CW1030 | *ATP11*_2 FISH-reverse | TAATACGACTCACTATAGGGAGAGTTCTTTCAGGGGGTCGAT |
| CW1031 | *ATP11*_3 FISH-forward | TGGATGTTGGAAAACTGAAGG |
| CW1032 | *ATP11*_3 FISH-reverse | TAATACGACTCACTATAGGGAGCTTTTTGTGCCCATCTTGCT |
| CW1033 | *ATP11*_4 FISH-forward | ATGGCAAATGCCAGGAATAA |
| CW1034 | *ATP11*_4 FISH-reverse | TAATACGACTCACTATAGGGAGGTTCGGTTTTGCATCCTCAG |
| CW1035 | *ATP11*_5 FISH-forward | GGAATTTGCAAGGCCACATA |
| CW1036 | *ATP11*_5 FISH-reverse | TAATACGACTCACTATAGGGAGGGTTCAACATGGCCATTCAT |
| CW1037 | *ATP11*_6 FISH-forward & qPCR-forward | TATGGTGCAATGGGTGAAGA |
| CW1519 | *ATP11* 3’UTRf | CGAAGAATTACTTCCGTATAC |
| CW1528 | *ATP11* 3’UTR 500r | TAATACGACTCACTATAGGGAGTAGGGCTTTTTTGATCATCC |
| CW1038 | *ATP11*_6 FISH-reverse | TAATACGACTCACTATAGGGAGCCGGAGAGGCCTTAGAGAAA |
| CW976 | *ATP11* qPCR-reverse | TCAATTTCTCGACGGTGAATC |
| KT1005 | *ATP11* deletion forward | CAAGACTTGAAAATAGAGAACAAAAGAGATTTAAGAATAACTATGCAGCTGAAGCTTCGTACGC |
| KT1006 | *ATP11* deletion reverse | TATATATATATATATATATACGTATACGGAAGTAATTCTTCGTTAGCATAGGCCACTAGTGGATCTG |
| CW995 | *ILM1*_1 FISH-forward | CAAGCCTTGAACTCCACCAA |
| CW996 | *ILM1*_1 FISH-reverse | TAATACGACTCACTATAGGGAGACAAAAGAAGGCGATCGTGA |
| CW997 | *ILM1*_2 FISH-forward | GCAAGCGATGAATTTACCGC |
| CW998 | *ILM1*_2 FISH-reverse | TAATACGACTCACTATAGGGAGCAGAAGAGCGAAAAGACCCA |
| CW999 | *ILM1*_3 FISH-forward | TTCCAATCTGTTGTGCCAGT |
| CW1000 | *ILM1*_3 FISH-reverse | TAATACGACTCACTATAGGGAGTTGACTCCCAAAGGTACGAGA |
| CW1001 | *ILM1*_4 FISH-forward | TGCCATTAGGGAGGAGAAAA |
| CW1002 | *ILM1*_4 FISH-reverse | TAATACGACTCACTATAGGGAGTGGGGTTCTTCAATATCCTCAT |
| CW1003 | *ILM1*_5 FISH-forward | CCGTGAAGACTGAAACCACA |
| CW1004 | *ILM1*_5 FISH-reverse | TAATACGACTCACTATAGGGAGCTTTCCCATCTTCATCGTCA |
| CW969 | *ILM1* qPCR-forward | CAATTGGGTCTTTTCGCTCT |
| CW970 | *ILM1* qPCR-reverse | CTGGCACAACAGATTGGAAG |
| CW1107 | *MRPL38*_1 FISH-forward | CGACAATTCAGGTGCACAAT |
| CW1108 | *MRPL38*_1 FISH-reverse | TAATACGACTCACTATAGGGAGCCAACCATTGCAGGACTCTT |
| CW1109 | *MRPL38*_2 FISH-forward | GCAAAGCCCTTGACTCAAAA |
| CW1110 | *MRPL38*_2 FISH-reverse | TAATACGACTCACTATAGGGAGACGACAATTGCGTGACAAAT |
| CW1111 | *MRPL38*_3 FISH-forward | ACCGTTGCATTCGGAGATAC |
| CW1112 | *MRPL38*_3 FISH-reverse | TAATACGACTCACTATAGGGAGTCTTGTCCCCAGAGGTTCAC |
| CW1113 | *MRPL38*_4 FISH-forward | TGGCTAATGATGGTTGTGTAGA |
| CW1114 | *MRPL38*_4 FISH-reverse | TAATACGACTCACTATAGGGAGACCCTACTTGCCAAAGAGCA |
| CW993 | *MRPL38* qPCR-forward | GTGAACCTCTGGGGACAAGA |
| CW994 | *MRPL38* qPCR-reverse | ACCCTACTTGCCAAAGAGCA |
| CW1450 | *MRPL38* pOMf | TGAAAG ACAAGGGATA CAATAAGATA TGCTCTTTGG CAAGTAGGGT CATATGCAGGTCGACAACCCTTAAT |
| CW1451 | *MRPL38* pOMr | TCCCTTGATTATAACGTTAATGAATGGAAAATGGGGGTACTTTTATTCTTAGCGGCCGCATAGGCCACT |
| CW880 | *AIM2*_1 FISH-forward | AGTTTGTCACGATGGAACACC |
| CW881 | *AIM2*_1 FISH-reverse | TAATACGACTCACTATAGGGAGGGGAGATGTAGAGCCTGCTG |
| CW882 | *AIM2*_2 FISH-forward | TGTGTATGGCAATAAATTCAACAA |
| CW883 | *AIM2*_2 FISH-reverse | TAATACGACTCACTATAGGGAGCCATGTACCCAGCACTAGCA |
| CW884 | *AIM2*_3 FISH-forward | CGATGCTATCTCATCGGACA |
| CW885 | *AIM2*_3 FISH-reverse | TAATACGACTCACTATAGGGAGTCTTGGTGACTTCAGGAGAATG |
| CW886 | *AIM2*_4 FISH-forward | TTTGCCGTCCAACACATTAG |
| CW887 | *AIM2*_4 FISH-reverse | TAATACGACTCACTATAGGGAGCGAAAGATGGATGTGCAATG |
| CW888 | *AIM2*_5 FISH-forward | GCAATTGATAGCAAGAAACCAA |
| CW889 | *AIM2*_5 FISH-reverse | TAATACGACTCACTATAGGGAGGTTTGCCGGAAAGATGTGAT |
| CW890 | *AIM2*_6 FISH-forward | CTCTTCAGTGGTGTGGCTCA |
| CW1179 | *AIM2* qPCR-forward | TTTGCCGTCCAACACATTAG |
| CW1180 | *AIM2* qPCR-reverse | CGAAAGATGGATGTGCAATG |
| JW270 | *RPL37b* qPCR-forward | ATTCGGTAAGCGTCACAA |
| JW271 | *RPL37b* qPCR-reverse | CCACAAGAGGAACAGGTC |
| SH809 | *hRLuc* qPCR-forward | TCATATCGCCTCCTGGAT |
| SH810 | *hRLuc* qPCR-reverse | CTTGTCTTGGTGCTCGTA |
| SH732 | Xrn1 S1 deletion-forward | TCAACACTTGTAACAACAGCAGCAACAAATATATATCAGTACGGTCAGCTGAAGCTTCGTACGC |
| SH733 | Xrn1 S2 deletion-reverse | ACTATTAAAGTAACCTCGAATATACTTCGTTTTTAGTCGTATGTTGCATAGGCCACTAGTGGATCTG |
| CW1149 | Puf5 S1 deletion-forward | TCTAC GCAAATTTAT AAATCAATTA CGATTTTTCC AGTTTCTCTT ATG CGTACGCTGCAGGTCGAC |
| CW1150 | Puf5 S2 deletion/tagging-reverse | TTTGTACAGTAAGAAGGAAAGAAAAAGAAAGAAAAAAAAGTATTA ATCGATGAATTCGAGCTCG |
| CW1151 | Puf5 S2 tagging-forward | CCATGAATAC CGCTAGAACA TCTGATGAAC TTCAATTCAC TTTGCCA CGTACGCTGCAGGTCGAC |
| CK504 | Puf3 pUG deletion-forward | TACGCATTTAAATTTCTTCTGAATAACGCAATATTGCGGGTATAACAGCTGAAGCTTCGTACG |
| CK505 | Puf3 pUG deletion-reverse | AAAAAAAAATAGTAAAAAGTGAAAGGAGAACGATGATAACACTAAGCATAGGCCACTAGTGGATCTG |
| CW1152 | Puf6 S1 deletion-forward | AGTA CTGAAATAAA GCACAATCAG GAATAACAAA TTAACTGACA ATG CGTACGCTGCAGGTCGAC |
| CW1153 | Puf6 S2 deletion-reverse | CAGATGCTTATATACCAAATATTGTGACTTTATCGTAGAAAATTTAATCGATGAATTCGAGCTCG |
| CW1342 | Sbp1 S1 deletion-forward | GAAGTTTCCCCCAAAAG AAAGAAGAAA ACCCTCAAAC GAAGAAAAAT ATGCGTACGCTGCAGGTCGAC |
| CW1343 | Sbp1 S2 deletion-reverse | AACTCTAGCAAAAACTCAAGTTAGAAATAGGGATGTGGGTAAGAAGTAATTAATCGATGAATTCGAGCTCG |
| CW1330 | Khd1 S1 deletion-forward | GCATCAACTTATCGGGTAAC TTAGAGACAG CATTAGTATA TATACCAGCC ATGCGTACGCTGCAGGTCGAC |
| CW1331 | Khd1 S2 deletion-reverse | GATAGTTTGTTTTGTCTGTGTGGGACGTGCGCACGCACACGTATATACTAATCGATGAATTCGAGCTCG |
| CW1333 | Pbp2 S1 deletion-forward | TCCAGCGCGGCATTAAAT AATCTTTCTG TAATACTCTT TAGCTCAATT ATGCGTACGCTGCAGGTCGAC |
| CW1334 | Pbp2 S2 deletion-reverse | GTTTCTGTATTTTTATTTTCTATGTGTTTTTATTGACTAGCAGTATATTTAATCGATGAATTCGAGCTCG |
| CW1339 | Ngr1 S1 deletion-forward | AGCTTTTTCATATCCTTCGC CATCGATTTT TGCCTGAAAA ATTTACACAA ATGCGTACGCTGCAGGTCGAC |
| CW1340 | Ngr1 S2 deletion-reverse | GATAAAACTGCGGACAAGATTAAAATTTTCTTTTTTTGTCTTTTGTAATCAATCGATGAATTCGAGCTCG |
| JW17 | Whi3 S1 deletion-forward | GCC TTT ATC GAT CAA TAT TTC AGA GGG AAA AAC CTG TAT CTC TTA GCA TGC GTA CGC TGC AGG TCG AC |
| JW16 | Whi3 S2 deletion-reverse | TAT AAT GTG ATA CAT GCA AGG AAA TCA GGT TTT TGC GGA ACC ATT TTT TTA ATC GAT GAA TTC GAG CTC G |
| CW1303 | Bsc1 S2 C-tagging-forward | GTAGTCACAGCGCTTGAACTAGTTGGGTCTTCTGAACTTGAACTTACTACTTAATCGATGAATTCGAGCTCG |
| CW1304 | Bsc1 S3 C-tagging-reverse | CGATG TAACCAGTTC AACCATTCAA ACTACTTCTG TTGATCCAAC CACTCGTACGCTGCAGGTCGAC |
| CW1257 | Atp11 S2 C-tagging-forward | TGTCATTAATATATATATATATATATATATACGTATACGGAAGTAATTCTTCGTTAATCGATGAATTCGAGCTCG |
| CW1256 | Atp11 S3 C-tagging-reverse | GGAT TCACCGTCGA GAAATTGATT TCGCTATCAC AGTCCATGGA AAATCGTACGCTGCAGGTCGAC |
| CW1394 | *HSP42* 3'UTR repl-forward | GAC GAAGAATTGG AGTTTGAAGAAAATCCCAAC CCTACGGTAG AAAATTGAGAGCTCGTTTTCGACACTGG |
| CW1395 | *HSP42* 3'UTR repl-reverse | TTATTCCGAGCAAGTCGATGAAGAAACCGCTTTTTGTTACAGTACAATGGTCCTTACCATTAAGTTGATC |
| CW1396 | *SEC59* 3'UTR repl-forward | TTTTGATACC TGCATTTATG ATGATTTGTG AAAAATTAAT TACTCTTTGAGAGCTCGTTTTCGACACTGG |
| CW1397 | *SEC59* 3'UTR repl-reverse | CCGTCAAAAGAAGGATGACGTTAAACCTGAATTGGCTAACAAAAGTGTGGGAGCTCGTTTTCGACACTGG |
| CW1399 | *YLR042C* 3'UTR repl-forward | GTCT TTACTTCGTT TTAATGTTAG AAACAATCGC TTATTTGTTT TCTTAAGAGCTCGTTTTCGACACTGG |
| CW1400 | *YLR042C* 3'UTR repl-reverse | TAAAAACTCCATGAAGAATGCCATGTCTGCATCTGCAGCTGCGGACCTGGTCCTTACCATTAAGTTGATC |
| CW1382 | *ATP11*-*SEC59* 3'UTR repl-forward | TTTTGATACC TGCATTTATG ATGATTTGTG AAAAATTAAT TACTCTTTGACGA AGAATTACTT CCGTATACGT |
| CW1383 | *ATP11*-*SEC59* 3'UTR repl-reverse | CCGTCAAAAGAAGGATGACGTTAAACCTGAATTGGCTAACAAAAGTGTGGAATACGTACCTTTGGGACCCAGA |
| CW1386 | *ATP11*-*YLR042C* 3'UTR repl-forward | GTCT TTACTTCGTT TTAATGTTAG AAACAATCGC TTATTTGTTT TCTTAACGA AGAATTACTT CCGTATACGT |
| CW1387 | *ATP11*-*YLR042C* 3'UTR repl-reverse | GTTCGTAACTGGATATCGAAATCACTTGATTAGAAACAATACGCCGGAATAATACGTACCTTTGGGACCCAGA |
| CW1388 | *BSC1*-*SEC59* 3'UTR repl-forward | TTTTGATACC TGCATTTATG ATGATTTGTG AAAAATTAAT TACTCTTTGAGTA GTAAGTTCAA GTTCAGAAG |
| CW1389 | *BSC1*-*SEC59* 3'UTR repl-reverse | CCGTCAAAAGAAGGATGACGTTAAACCTGAATTGGCTAACAAAAGTGTGGGCTACTTAGATCAGCTGAACTTG |
| CW1392 | *BSC1*-*YLR042C* 3'UTR repl-forward | GTCT TTACTTCGTT TTAATGTTAG AAACAATCGC TTATTTGTTT TCTTAAGTA GTAAGTTCAA GTTCAGAAG |
| CW1393 | *BSC1*-*YLR042C* 3'UTR repl-reverse | GTTCGTAACTGGATATCGAAATCACTTGATTAGAAACAATACGCCGGAATGCTACTTAGATCAGCTGAACTTG |
| CW1407 | *YLR042C*_1 FISH-forward | CGCCCCAATTGTGCTACTAC |
| CW1408 | *YLR042C*_1 FISH-reverse | TAATACGACTCACTATAGGGAGCACTTTCTGTCCAAGGGCAG |
| CW1409 | *YLR042C*_2 FISH-forward | TCATCTTCCAAGCACCACCT |
| CW1410 | *YLR042C*_2 FISH-reverse | TAATACGACTCACTATAGGGAGACGACGATGATGAAAAGCCG |
| CW1411 | *YLR042C*_3 FISH-forward | CCAAAAGTCACTTCCAGCGT |
| CW1412 | *YLR042C*_3 FISH-reverse | TAATACGACTCACTATAGGGAGCTCGACGAAGAAGGCAATGG |
| CW1413 | *YLR042C*_4 FISH-forward | GTTCAAGTACAAGCACAGGAGG |
| CW1414 | *YLR042C*_4 FISH-reverse | TAATACGACTCACTATAGGGAGACAAAGTAAAGACATGGCTTGAC |
| CW190 | *SEC59*_1 FISH-forward | GAAGCCAAATGCCAACTGAG |
| CW191 | *SEC59*_1 FISH-reverse | TAATACGACTCACTATAGGGAGCGCCCACATCAAATTTACGA |
| CW192 | *SEC59*_2 FISH-forward | TGGTGTGTTTGATAATGGTTGG |
| CW193 | *SEC59*_2 FISH-reverse | TAATACGACTCACTATAGGGAGCGAACTTGGGTAGGCTTTTG |
| CW194 | *SEC59*_3 FISH-forward | GATGGCGAACTCAGTTGGTAG |
| CW195 | *SEC59*_3 FISH-reverse | TAATACGACTCACTATAGGGAGCCTGTCACCAGCCTCAAAAT |
| CW196 | *SEC59*_4 FISH-forward | TTTCAGAGGCTGGCACAGTA |
| CW197 | *SEC59*_4 FISH-reverse | TAATACGACTCACTATAGGGAGGCAAGGATAATGCCCTTCAA |
| CW198 | *SEC59*_5 FISH-forward | CATCGGGCTTCCACTTTTT |
| CW199 | *SEC59*_5 FISH-reverse | TAATACGACTCACTATAGGGAGGCCAAAGTAGCGGATTTTCA |
| CW200 | *SEC59*_6 FISH-forward | AACACCTCCCGAAAATTGTG |
| CW201 | *SEC59*_6 FISH-reverse | TAATACGACTCACTATAGGGAGTGAATCCATTTGGAATGACG |
| CW202 | *SEC59*_7 FISH-forward | TGCTGATGATAGGGACCACA |
| CW203 | *SEC59*_7 FISH-reverse | TAATACGACTCACTATAGGGAGAAAGGTGTTGATATTCCGAAGAG |
| CW204 | *SEC59*_8 FISH-forward | TCGTTGGAAAGGTACACAAAAA |
| CW205 | *SEC59*_8 FISH-reverse | TAATACGACTCACTATAGGGAGCCAAACAAACGATAAAACTCG |
| CW1396 | *SEC59_*pCore_forward | TTTTGATACCTGCATTTATG ATGATTTGTG AAAAATTAATTACTCTTTGAGAGCTCGTTTTCGACACTGG |
| CW1397 | *SEC59_*pCore_reverse | CCGTCAAAAGAAGGATGACGTTAAACCTGAATTGGCTAACAAAAGTGTGGTCCTTACCATTAAGTTGATC |
| CW1399 | *YLR042C_*pCore_forward | GTCTTTACTTCGTT TTAATGTTAG AAACAATCGCTTATTTGTTT TCTTAAGAGCTCGTTTTCGACACTGG |
| CW1401 | *YLR042C*_pCore_reverse | GTTCGTAACTGGATATCGAAATCACTTGATTAGAAACAATACGCCGGAATTCCTTACCATTAAGTTGATC |
| CW1388 | *BSC1-SEC59* 300 3’_repl_forward | TTTTGATACC TGCATTTATG ATGATTTGTG AAAAATTAAT TACTCTTTGAGTA GTAAGTTCAA GTTCAGAAG |
| CW1389 | *BSC1-SEC59* 300 3’_repl_reverse | CCGTCAAAAGAAGGATGACGTTAAACCTGAATTGGCTAACAAAAGTGTGGGCTACTTAGATCAGCTGAACTTG |
| CW1382 | *ATP11-SEC59* 300 3’_repl_forward | TTTTGATACC TGCATTTATG ATGATTTGTG AAAAATTAAT TACTCTTTGACGA AGAATTACTT CCGTATACGT |
| CW1383 | *ATP11-SEC59* 300 3’_repl_reverse | CCGTCAAAAGAAGGATGACGTTAAACCTGAATTGGCTAACAAAAGTGTGGAATACGTACCTTTGGGACCCAGA |
| CW1392 | *BSC1-YLR042C* 300 3’_repl_forward | GTCTTTACTTCGTTTTAATGTTAG AAACAATCGCTTATTTGTTTTCTTAAGTAGTAAGTTCAAGTTCAGAAG |
| CW1393 | *BSC1-YLR042C* 300 3’_repl_reverse | GTTCGTAACTGGATATCGAAATCACTTGATTAGAAACAATACGCCGGAATGCTACTTAGATCAGCTGAACTTG |
| CW1386 | *ATP11-YLR042C* 300 3’_repl_forward | GTCTTTACTTCGTTTTAATGTTAGAAACAATCGC TTATTTGTTTTCTTAACGAAGAATTACTT CCGTATACGT |
| CW1387 | *ATP11-YLR042C* 300 3’_repl_reverse | GTTCGTAACTGGATATCGAAATCACTTGATTAGAAACAATACGCCGGAATAATACGTACCTTTGGGACCCAGA |
| CW546 | *BSC1* MS2 forward | TAACCAGTTCAACCATTCAAACTACTTCTGTTGATCCAACCACTTAAGCCGCTCTAGAACTAGTGGATCC |
| CW547 | *BSC1* MS2 reverse | AGTCACAGCGCTTGAACTAGTTGGGTCTTCTGAACTTGAACTTACTACGCATAGGCCACTAGTGGATCTG |
| CW662 | *PTP3*_1 FISHf | CGGTGTTATTGAAGAGGGCG |
| CW663 | *PTP3*_1 FISHr | TAATACGACTCACTATAGGGAGGAACAACCCTGCGGCTTATT |
| CW664 | *PTP3*_2 FISHf | TGTATGTTTGCCTTCCACGC |
| CW665 | *PTP3*_2 FISHr | TAATACGACTCACTATAGGGAGGATTTCAACACGCTTTGCTCG |
| CW666 | *PTP3*_3 FISHf | GCGACAACACCACTATCGTC |
| CW667 | *PTP3*_3 FISHr | TAATACGACTCACTATAGGGAGCGGAGAGGGAGAACTGGAAA |
| CW668 | *PTP3*_4 FISHf | CCTGAATGGTTCCAGCATCTG |
| CW669 | *PTP3*_4 FISHr | TAATACGACTCACTATAGGGAGCAGAATGGTTCAAGCGCCTT |
| CW670 | *PTP3*_5 FISHf | AAATCGATGCTCAGCCTTGA |
| CW671 | *PTP3*_5 FISHr | TAATACGACTCACTATAGGGAGGGCTCCTAATTCAACGCCTG |
| CW672 | *PTP3*_6 FISHf | CACAACAAGAAGAGTGCGCT |
| CW673 | *PTP3*_6 FISHr | TAATACGACTCACTATAGGGAGTCAATGAGAGAACCAACGGAAC |
| CW674 | *PTP3*_7 FISHf | AGAGAACGGATGGTGATGGA |
| CW675 | *PTP3*_7 FISHr | TAATACGACTCACTATAGGGAGCATCCACGAATGTCTGCAAGA |
| CW654 | *NSL1*_1 FISHf | TGTTGAGCAGCTTCGAAGTA |
| CW655 | *NSL1*_1 FISHr | TAATACGACTCACTATAGGGAGCCTCTCTCCTAACAGCGTCA |
| CW656 | *NSL1*_2 FISHf | GACACGGTAGGAAAGACGGT |
| CW657 | *NSL1*_2 FISHr | TAATACGACTCACTATAGGGAGTGCTCATTCAGGTCAAGGTCA |
| CW658 | *NSL1*_3 FISHf | GGCACAATTGGATGGGAGAA |
| CW659 | *NSL1*_3 FISHr | TAATACGACTCACTATAGGGAGCGGCATCGTCGGTACTATCA |
| CW660 | *NSL1*_4 FISHf | GCAGGATTACGTTGCCTCAC |
| CW661 | *NSL1*_4 FISHr | TAATACGACTCACTATAGGGAGGTCCATTAAGCGCTTGACCT |
| JW01 | Pub1-Tag S2 primer | CCGGTTATGTCTGAGCAACAACAGCAACAGCAGCAACAGCAGCAACAACAACGTACGCTGCAGGTCGAC |
| JW02 | Pub1-Tag S3 primer | GCCTCTCTTTCTTCTTTCTTTTTGTTTCATTCCACTTTTCTTCATAATATTTAATCGATGAATTCGAGCTCC |
| JW024 | Tif4632 S2 primer | GAATTAAAAAGGAAAAAGACTAGCTTATCGTTTCTAAAAGAAAATCTTTTAATCGATGAATTCGAGCTCG |
| JW025 | Tif4632 S3 primer | CCAAGAGCTAATATGTTCGACGCATTAATGAATAACGATGGGGACAGTGATCGTACGCTGCAGGTCGAC |
| CW1534 | U1AfGFP-gib-F | TGCTTTTCAAGATACCCGGATCATATGAAACGGCATGACTTTTTCAAG |
| CW1535 | U1AfGFP-gib-R | TCCACCTCCACCTCCGCAGCCGGATCCTTTGTATAG |
| CW1536 | PUF5-gib-F | ATCCGGCTGCGGAGGTGGAGGTGGAGGTGGAGGTATGGTATGTATTATCTCTTAATTCC |
| CW1537 | PUF5-gib-R | AAGGGAACAAAAGCTGGTACCTTATGGCAAAGTGAATTGAAG |
| CW1538 | PUF3-gib-F | ATCCGGCTGCGGAGGTGGAGGTGGAGGTGGAGGTATGGAAATGAACATGGATATGG |
| CW1539 | PUF3-gib-R | AAGGGAACAAAAGCTGGTACCTCACACCTCCGCATTTTC |
| CW1543 | pU1A-gib-R | ATCCGGGTATCTTGAAAAG |
| CW1544 | pU1A-gib-F | GTACCAGCTTTTGTTCCCTTAGCTTTTGTTCCCTTTAG |
| CW43 | STL1-3'for-SpeI | ATTACTAGTCACGTTGGCTGATTTCTCAA |
| CW44 | STL1-3'rev-NotI | ATTGCGGCCGCCGTCGTCATAAGAGCCCAAT |
| CW1540 | DCP1-pUG deletion forward | ATAAGCATTTATCTTTGCAACACATCACAAGAAAAGCTGTGCACACAGCTGAAGCTTCGTACG |
| CW1541 | DCP1-pUG deletion reverse | TCATTATTTAAAAAAAATTCTCACTTGGGCATCTCACCTCTGTGCGCATAGGCCACTAGTGGATCTG |
