# Supplementary material for: Context-dependent deposition and regulation of mRNAs in P-bodies
Source: eLife. 2018 Jan 3;7:e29815. doi: 10.7554/eLife.29815 (PMC5752201; doi:10.7554/eLife.29815)
Supplement: Supplementary file 5. [file elife-29815-supp5.docx]

**Supplemental File 5. List of strains used in this study.**

| Strain ID | Designation | Genotype | Reference |
| --- | --- | --- | --- |
| NYY0-1 | ARF1 | *MAT a ade2::ARF1::ADE2 arf1::HIS3 arf2::HIS3 ura3 lys2 trp1 his3 leu2* | Yahara et al., 2001 |
| YAS1936 | Dcp2-HBH | *MAT a ade2::ARF1::ADE2 arf1::HIS3 arf2::HIS3 ura3 lys2 trp1 his3 leu2 DCP2::DCP2-HBH-kanMX4* | Weidner et al., 2014 |
| YAS3240 | Scd6-HBH | *MAT a ade2::ARF1::ADE2 arf1::HIS3 arf2::HIS3 ura3 lys2 trp1 his3 leu2 SCD6::SCD6-HBH-TRP1* | Weidner et al., 2014 |
| YAS1031A | Dcp2-GFP | *MAT a ade2::ARF1::ADE2 arf1::HIS3 arf2::HIS3 ura3 lys2 trp1 his3 leu2 DCP2::DCP2-yEGFP-kanMX4* | Kilchert et al., 2010 |
| YAS2789 | Dcp2-3HA | *MAT a ade2::ARF1::ADE2 arf1::HIS3 arf2::HIS3 ura3 lys2 trp1 his3 leu2 DCP2::DCP2-3HA-klTRP1* | This study |
| YAS4381 | Δ*xrn1* | *MAT a ade2::ARF1::ADE2 arf1::HIS3 arf2::HIS3 ura3 lys2 trp1 his3 leu2 xrn1::natNT2* | This study |
| YAS2491 | Δ*puf3* | *MAT a ade2::ARF1::ADE2 arf1::HIS3 arf2::HIS3 ura3 lys2 trp1 his3 leu2 puf3::klLEU2* | This study |
| YAS4962 | Δ*puf5* | *MAT a ade2::ARF1::ADE2 arf1::HIS3 arf2::HIS3 ura3 lys2 trp1 his3 leu2 puf5::natNT2* | This study |
| YAS4469 | Dcp2-GFP Δ*puf5* | *MAT a ade2::ARF1::ADE2 arf1::HIS3 arf2::HIS3 ura3 lys2 trp1 his3 leu2 DCP2::DCP2-yEGFP-kanMX4 puf5::natNT2* | This study |
| YAS4610 | Dcp2-GFP Δ*puf3* | *MAT a ade2::ARF1::ADE2 arf1::HIS3 arf2::HIS3 ura3 lys2 trp1 his3 leu2 DCP2::DCP2-yEGFP-kanMX4 puf3::klLEU2* | This study |
| YAS4470 | Dcp2-GFP Δ*puf6* | *MAT a ade2::ARF1::ADE2 arf1::HIS3 arf2::HIS3 ura3 lys2 trp1 his3 leu2 DCP2::DCP2-yEGFP-kanMX4 puf6::natNT2* | This study |
| YAS4543 | Dcp2-GFP Δ*sbp1* | *MAT a ade2::ARF1::ADE2 arf1::HIS3 arf2::HIS3 ura3 lys2 trp1 his3 leu2 DCP2::DCP2-yEGFP-kanMX4 sbp1::natNT2* | This study |
| YAS4544 | Dcp2-GFP Δ*khd1* | *MAT a ade2::ARF1::ADE2 arf1::HIS3 arf2::HIS3 ura3 lys2 trp1 his3 leu2 DCP2::DCP2-yEGFP-kanMX4 khd1::natNT2* | This study |
| YAS4546 | Dcp2-GFP Δ*pbp2* | *MAT a ade2::ARF1::ADE2 arf1::HIS3 arf2::HIS3 ura3 lys2 trp1 his3 leu2 DCP2::DCP2-yEGFP-kanMX4 pbp2::natNT2* | This study |
| YAS4843 | Dcp2-GFP Δ*ngr1* | *MAT a ade2::ARF1::ADE2 arf1::HIS3 arf2::HIS3 ura3 lys2 trp1 his3 leu2 DCP2::DCP2-yEGFP-kanMX4 ngr1::natNT2* | This study |
| YAS4472 | Dcp2-GFP Δ*whi3* | *MAT a ade2::ARF1::ADE2 arf1::HIS3 arf2::HIS3 ura3 lys2 trp1 his3 leu2 DCP2::DCP2-yEGFP-kanMX4 whi3::natNT2* | This study |
| YAS4628 | Dcp2-GFP Δ*atp11* | *MAT a ade2::ARF1::ADE2 arf1::HIS3 arf2::HIS3 ura3 lys2 trp1 his3 leu2 DCP2::DCP2-yEGFP-kanMX4 atp11::URA3* | This study |
| YAS4629 | Dcp2-GFP Δ*bsc1* | *MAT a ade2::ARF1::ADE2 arf1::HIS3 arf2::HIS3 ura3 lys2 trp1 his3 leu2 DCP2::DCP2-yEGFP-kanMX4 bsc1::URA3* | This study |
| YAS4547 | Dcp2-GFP  Bsc1-3HA | *MAT a ade2::ARF1::ADE2 arf1::HIS3 arf2::HIS3 ura3 lys2 trp1 his3 leu2 DCP2::DCP2-yEGFP-kanMX4 BSC1::BSC1-3HA-klTRP1* | This study |
| YAS4551 | Dcp2-GFP  Atp11-3HA | *MAT a ade2::ARF1::ADE2 arf1::HIS3 arf2::HIS3 ura3 lys2 trp1 his3 leu2 DCP2::DCP2-yEGFP-kanMX4 ATP11::ATP11-3HA-klTRP1* | This study |
| YAS4650 | Dcp2-3HA *SEC59*-*BSC1*(3’UTR) | *MAT a ade2::ARF1::ADE2 arf1::HIS3 arf2::HIS3 ura3 lys2 trp1 his3 leu2 DCP2::DCP2-3HA-klTRP1*  *SEC59::SEC59-BSC1(3’UTR)* | This study |
| YAS4651 | Dcp2-3HA *SEC59*-*ATP11*(3’UTR) | *MAT a ade2::ARF1::ADE2 arf1::HIS3 arf2::HIS3 ura3 lys2 trp1 his3 leu2 DCP2::DCP2-3HA-klTRP1*  *SEC59::SEC59-ATP11(3’UTR)* | This study |
| YAS4659 | Dcp2-3HA *YLR042C*-*BSC1*(3’UTR) | *MAT a ade2::ARF1::ADE2 arf1::HIS3 arf2::HIS3 ura3 lys2 trp1 his3 leu2 DCP2::DCP2-3HA-klTRP1*  *YLR042C:: YLR042C-BSC1(3’UTR)* | This study |
| YAS4660 | Dcp2-3HA *YLR042C*-*ATP11*(3’UTR) | *MAT a ade2::ARF1::ADE2 arf1::HIS3 arf2::HIS3 ura3 lys2 trp1 his3 leu2 DCP2::DCP2-3HA-klTRP1*  *YLR042C:: YLR042C-ATP11(3’UTR)* | This study |
| YAS4163 | Dcp2-2mcherry | *MAT a ade2::ARF1::ADE2 arf1::HIS3 arf2::HIS3 ura3 lys2 trp1 his3 leu2 DCP2::DCP2-2xyemcherry-hphNT1* | This study |
| YAS4457 | Dcp2-2mcherry  Puf5-GFP | *MAT a ade2::ARF1::ADE2 arf1::HIS3 arf2::HIS3 ura3 lys2 trp1 his3 leu2 DCP2::DCP2-2xyemcherry-hphNT1*  *PUF5::PUF5-yeGFP- klTRP1* | This study |
| YAS4675 | Dcp2-2mcherry  *BSC1*-24xMS2SL | *MAT a ade2::ARF1::ADE2 arf1::HIS3 arf2::HIS3 ura3 lys2 trp1 his3 leu2 DCP2::DCP2-2xyemcherry-hphNT1*  *BSC1::BSC1*-24xMS2SL | This study |
| YAS4850 | Rlm1-9myc | *MAT a ade2::ARF1::ADE2 arf1::HIS3 arf2::HIS3 ura3 lys2 trp1 his3 leu2 RLM1::RLM1-9myc* | This study |
| YAS4851 | Mrpl38-9myc | *MAT a ade2::ARF1::ADE2 arf1::HIS3 arf2::HIS3 ura3 lys2 trp1 his3 leu2 MRPL38::MRPL38-9myc* | This study |
| YAS4854 | wild-type(ARF1)+  p416GPD-ATP11 | *MAT a ade2::ARF1::ADE2 arf1::HIS3 arf2::HIS3 ura3 lys2 trp1 his3 leu2*  *+*p416GPD-ATP11 (*URA3*) | This study |
| YAS4855 | Dcp2-GFP Δ*puf5*+  p416GPD-ATP11 | *MAT a ade2::ARF1::ADE2 arf1::HIS3 arf2::HIS3 ura3 lys2 trp1 his3 leu2 DCP2::DCP2-yEGFP-kanMX4 puf5::natNT2*  *+*p416GPD-ATP11 (*URA3*) | This study |
| YAS3005 | Tif4632-GFP | *MAT a ade2::ARF1::ADE2 arf1::HIS3 arf2::HIS3 ura3 lys2 trp1 his3 leu2 TIF4632:: TIF4632-yeGFP- klTRP1* | This study |
| YAS2236 | Pub1-GFP | *MAT a ade2::ARF1::ADE2 arf1::HIS3 arf2::HIS3 ura3 lys2 trp1 his3 leu2 PUB1:: PUB1-yeGFP- klTRP1* | This study |
| YAS4983 | Dcp2-2mcherry+PGK1-U1A-STL1+U1A-GFP-Puf3 | *MAT a ade2::ARF1::ADE2 arf1::HIS3 arf2::HIS3 ura3 lys2 trp1 his3 leu2 DCP2::DCP2-2xyemcherry-hphNT1*  *+* pRS2037-STL1+pRP1187-Puf3 | This study |
| YAS4984 | Dcp2-2mcherry+PGK1-U1A-STL1+U1A-GFP-Puf5 | *MAT a ade2::ARF1::ADE2 arf1::HIS3 arf2::HIS3 ura3 lys2 trp1 his3 leu2 DCP2::DCP2-2xyemcherry-hphNT1*  *+* pRS2037-STL1+pRP1187-Puf5 | This study |
| YAS4963 | Δ*dcp1* | *MAT a ade2::ARF1::ADE2 arf1::HIS3 arf2::HIS3 ura3 lys2 trp1 his3 leu2 dcp1::klLEU2* | This study |
| YAS4964 | Δ*puf5*Δ*dcp1* | *MAT a ade2::ARF1::ADE2 arf1::HIS3 arf2::HIS3 ura3 lys2 trp1 his3 leu2 puf5::natNT2 dcp1::klLEU2* | This study |
| YAS4985 | Dcp2-GFP Δ*dcp1* | *MAT a ade2::ARF1::ADE2 arf1::HIS3 arf2::HIS3 ura3 lys2 trp1 his3 leu2 DCP2::DCP2-EGFP-kanMX4 dcp1::klLEU2* | This study |
| YAS4986 | Dcp2-GFP Δ*puf5*Δ*dcp1* | *MAT a ade2::ARF1::ADE2 arf1::HIS3 arf2::HIS3 ura3 lys2 trp1 his3 leu2 DCP2::DCP2-EGFP-kanMX4 puf5::natNT2 dcp1::klLEU2* | This study |
